# Resistive switching behavior of reduced graphene oxide memory cells for low power nonvolatile device application

**DOI:** 10.1038/srep26763

**Published:** 2016-05-31

**Authors:** Sangram K. Pradhan, Bo Xiao, Saswat Mishra, Alex Killam, Aswini K. Pradhan

**Affiliations:** 1 Center for Materials Research, Norfolk State University, 700 Park Ave., Norfolk, VA 23504, USA.

## Abstract

Graphene Oxide (GO) based low cost flexible electronics and memory cell have recently attracted more attention for the fabrication of emerging electronic devices. As a suitable candidate for resistive random access memory technology, reduced graphene oxide (RGO) can be widely used for non-volatile switching memory applications because of its large surface area, excellent scalability, retention, and endurance properties. We demonstrated that the fabricated metal/RGO/metal memory device exhibited excellent switching characteristics, with on/off ratio of two orders of magnitude and operated threshold switching voltage of less than 1 V. The studies on different cell diameter, thickness, scan voltages and period of time corroborate the reliability of the device as resistive random access memory. The microscopic origin of switching operation is governed by the establishment of conducting filaments due to the interface amorphous layer rupturing and the movement of oxygen in the GO layer. This interesting experimental finding indicates that device made up of thermally reduced GO shows more reliability for its use in next generation electronics devices.

The rapid expansion of nonvolatile memory technology has enabled a revolution of digital technology due to its higher density, more speed and greater scalability. High output resistive random access devices are fabricated by overcoming the drawback shown by the traditional Si based device^1^,^2^,^3^. Thin insulating metal oxide layer can be actively used to develop efficient memory devices with superior electrical performance^4^,^5^. However, these devices are inefficient to offer the transparency and flexibility due to the materials property. The carbon family materials, such as fullerenes, carbon nanotubes and graphene are being explored as an alternative resistive layered material for nonvolatile memory application because of their flexibility and transparecy^6^,^7^. Specifically, instability in the switching processes like set, reset and endurance behavior of the device are the concern factors to obtain multilevel operation of the transparent and flexible devices. However, detail studies on transparent resistive random access memory (ReRAM) devices are important for next generation technology, and are quite deficient considering the opacity of Si, a promising semiconductor for major ReRAM application until now^8^. Chemical derivative of graphite well known as graphene oxide (GO), has drawn significant attention for exciting physical, structural, and chemical characteristic. It consists of hydrophilic oxygenated transparent flexible graphene sheet having potential application for high integration density and fast memory application^9^. Moreover; the physical mechanism behind the resistive switching behavior in thermally reduced graphene oxide (RGO) has not been studied in detail for exploiting this material to its full extent. Interestingly, presence of the one-carbon atom cationic state in reduced GO leads to shift the material to a lower electrical resistance state (LRS). Similarly, chemically removing oxygen ions from GO changes to a high resistive state (HRS), which is a primary concern for the device degradation^3^.

This paper reports the detail study based on the structural, optical and resistive switching characteristics of thermally reduced GO and the electrode dependence switching property of small power consuming GO memory cell and their reliability as resistive random access memory. The studies on different cell diameter and thickness, different scan voltages and for different period of time are pursued to prove the reliability as resistive random access memory.

## Results and Discussion

**Figure 1:**
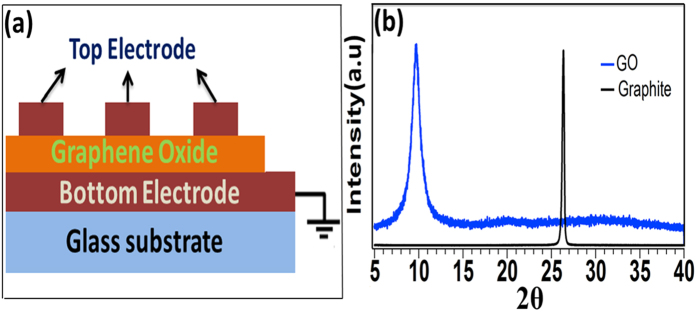
(**a**) Schematic for the Metal/GO/Metal structure on glass substrate. (**b**) XRD structure of graphite and GO.

Figure 1a represents the schematic diagram of the resistive memory device consists of reduced graphene oxide sandwich between two metal electrodes to make Metal-Insulator –Metal (MIM) structure. The thickness (33 nm) of RGO layer is shown in the supplementary Figure S1. The clear brown color of GO suspension is a characteristic feature of suspended GO in highly oxidized form and thermally RGO dispersions at various temperatures are shown in supplementary Figure S2. Powder x-ray diffraction patterns of graphite powders and GO are shown in Fig. 1b. A sharp and strong peak at 2θ = 26.40° value observed from the XRD spectra indicates a highly ordered structure of graphite powder. However, (001) reflection peak of fully oxidized GO pattern at 2θ = 10.21° represents the existence of intercalated H_2_O molecule and oxygenated functional group which is strongly attached to the GO. The XRD spectra of thermally reduced graphene oxide for different temperature are shown in the supplementary Figure S3.

**Figure 2:**
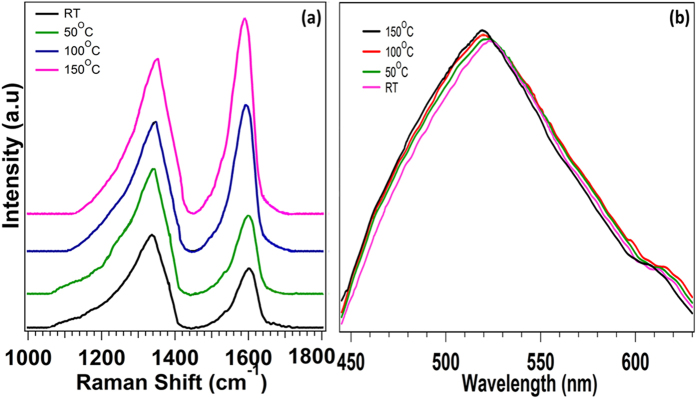
(**a**) Raman Spectra (**b**) PL spectra of GO reduced at different temperature.

Figure 2a shows the Raman spectrum of GO and thermally reduced GO in the frequency range of 1000–1800 cm^−1^. Observation of two distinct broad peak around 1332 and 1589 cm^−1^ is the characteristic of disorder induced D band with higher relative intensity and blue shift G band of GO, respectively. The amorphous carbon contains certain fraction of sp^3^ carbon lattice that is associated with strong disruption of its structure in chemically synthesized GO due to its oxidative nature. However, both the G and D bands of amorphization of graphite undergo significant changes with very small shifting of their peak position during low temperature thermal reduction. The D band shows blue shift while G bands shows red shift with increasing in intensity that is attributed to self-healing of graphite by gradual removal of oxygen as well as other functional groups from partial reduced GO. This is similar to the observation of other groups^10^,^11^ where formation of sharp G peak suppresses the intensity of the D peak in thermally reduced graphitic oxide. The PL spectra of thermally reduced GO indicate the observation of a blue shift in PL spectra of GO annealed at different temperatures as shown in Fig. 2b, and is attributed to the formation of confined graphene domain in GO^12^,^13^. I-V graph and FTIR spectra of thermally reduced graphene oxide at different temperature are shown in supplementary Figures S4 and S5.

**Figure 3:**
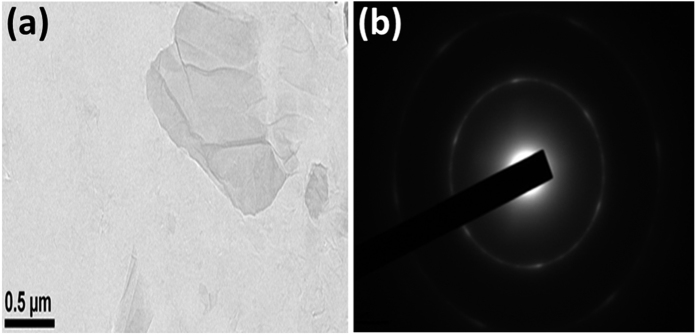
(**a**) TEM image of thermally (100 °C) RGO sheet at (**b**) Electron diffraction pattern of few layers thick thermally (100 °C) RGO thin film.

For further structural analysis of GO sheets, transmission electron microscopy (TEM) is employed to investigate the physical structure of GO sheets. TEM image and its diffraction pattern were shown in Fig. 3. It is noticed that GO sheets are all most electron transparent in nature from its low magnification TEM image as shown in Fig. 3a. However, different contrast of visibility of GO image around the top corner area of the figure contains a few folds of GO sheets which consist of few layers. Figure 3b represents the selected area electron diffraction pattern of GO sheet. Individual diffraction spots are not visible in GO sheet and the contributing array merge into a ring pattern. This image indicates that GO is not completely amorphous in nature. The six fold diffraction pattern of observed few layers thick GO is made up with hexagonal lattice and is the characteristic of polycrystalline nature of the materials. FESEM images of graphite powder and thermally reduced GO annealed at 150 °C are shown in supplementary Figure S6.

**Figure 4:**
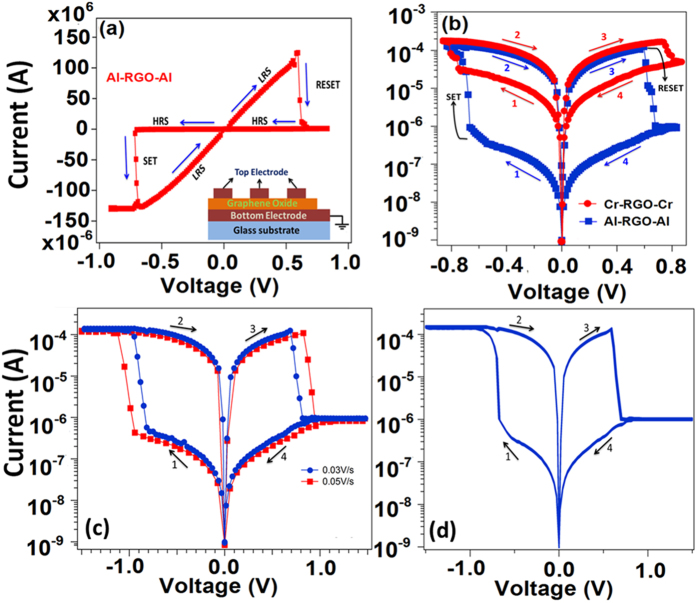
(**a**) *I*-*V* characteristics of Al/RGO/Al structure. The arrows indicate the sweep direction (**b**) *I*-*V* characteristics in semilogarithmic scale of Al/RGO/Al and Cr/RGO/Cr structure (**c**) I-V characteristic of Al/GO/Al resistive switching device for different scan voltage (d) 25 experimental switching I-V curves of the device for different period of time.

Current-voltage characteristic of Al/RGO/Al resistive switching device is shown in Fig. 4a. The voltage sweeps from 0 V → −0.9 → 0 V → 0.9 V → 0 V sequence at a rate of 0.015 V/s during the measurement. With increasing negative voltage, the current starts increasing and jumps suddenly at 0.68 V. This characteristic of the resistive switching device is said to be “Set” process where the device switches to a lower resistance state (LRS or “on” state) from higher resistance state (HRS or off state). During this Set process, we fix the current compliance value of 5 mA to prevent the sample from its permanent electrical breakdown. During voltage sweeping from −0.9 V to +0.6 V, the device withstand its LRS and further increase in voltage (0.62 V) favors the device quickly moves to a HRS from its previous LRS. This state of the device is said to be “Reset” process and the device shows this behavior till next “Set” process is achieved. To study further, the semi logarithmic scale I-V curves of RGO RRAM comprised of 33 nm thick of GO with Al/RGO/Al and Cr/RGO/Cr structure are shown in Fig. 4b. Both devices perform the bipolar resistive switching behavior with a stable resistive switching characteristic between the HRS and the LRS which is observed in Al/RGO/Al device. The device shows the LRS to HRS (on/off) current ration of ~10^2^ and very small set/reset voltages range of 0.6 V. However, a small value of “on/off” ratio is observed in Cr/RGO/Cr device at a similar set/reset voltage of 0.62 V. Furthermore, I-V characteristic for different sweep steps (0.03 and 0.05 V/s) of 33 nm thick GO film is shown in Fig. 4c. By changing the different voltage sweep step, the SET and RESET voltage of the device increases towards higher voltage. Figure 4d shows the 25 experimental switching I-V curves of the Al/GO/Al resistive switching device for different cycle with increase in period of time for few minutes to hours. These I-V graphs do not change much with increase in time and cycle resulting to the high degree of stability and repeatability. Current-voltage behavior of Al/RGO/Al resistive switching device with 50 nm GO film thickness is shown in supplementary Figure S7.

**Figure 5:**
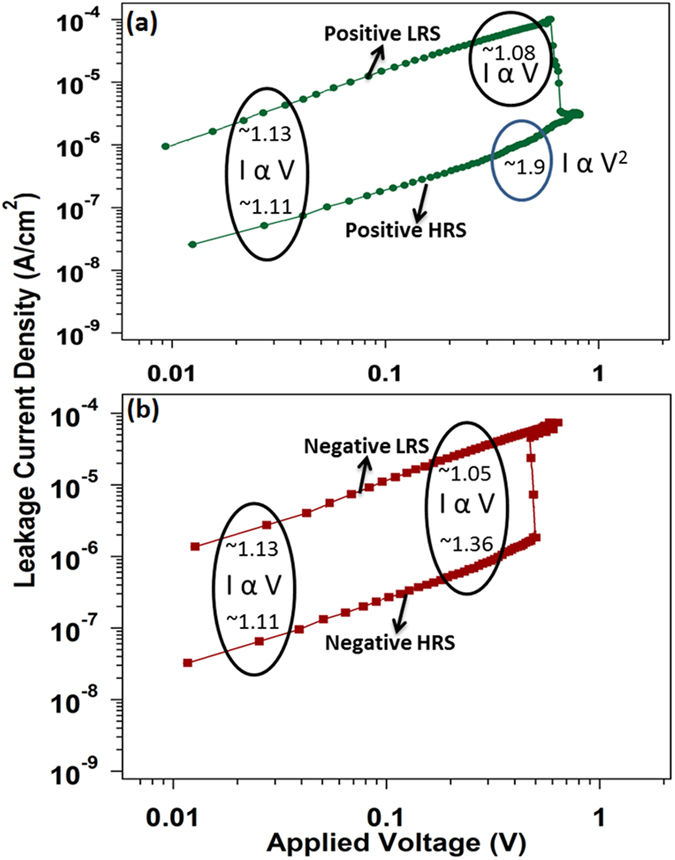
The current(I) – voltage(V) curves of the Al/RGO/Al device in a double logarithmic scale under four different situations of the memory operation. The indicated numbers mean the slope value of log I – log V curve.

Figure 5 shows the current-voltage graph plotted in a double logarithm scale to study the switching behavior under four different situations of memory operation. During the characterization of resistive random access memory device, the Ohmic behavior is described by I(V) = αV while the relationship I(V) = αV + βV^2^ is the representation of space charge limited conduction mechanism^14^,^15^. The numbers indicated in the graph show the values of slope of the curve. The slope of the graph shows the values of almost 1 in entire +ve polarity high resistance state before shifting to “set” operation state with a slope value of roughly 2. This type of switching behavior from the I-V is governed by the space charge limited conduction mechanism from LRS to HRS state with a value of slope 2 where the dielectric layer is triggered by the deficiency of oxygen. Therefore the formation of electron traps and oxygen vacancies take place easily in the GO layer which favors the generation of conducting path resulting in change of the resistive state of the device^16^.

**Figure 6:**
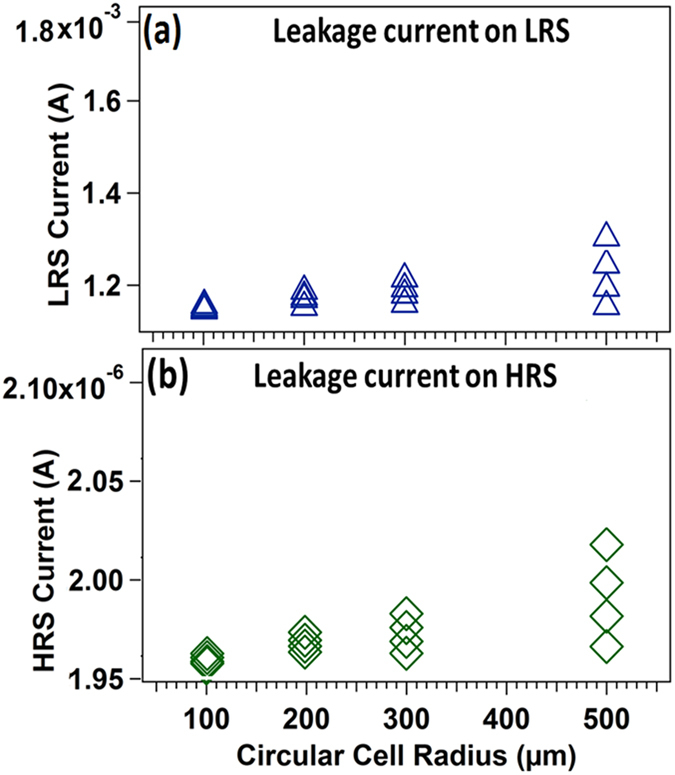
Cell area dependent of leakage current characteristic in Al/RGO/Al resistive memory device (**a**) LRS and (**b**) HRS state.

Although, the BRS behavior of reduced GO takes place through two different ways; the oxygen migration in RGO layer and diffusion of metal from electrode. To understand clearly, a further study was carried out to confirm the real mechanism of the switching operation. To further analyze, circular patterns 100, 200, 300, and 500 μm diameter of four different conducting circular areas were fabricated. The leakage current is reported to be dependent on the cell size if the leakage current in the device is governed by the oxygen migration^17^. From Fig. 6, the graph shows that the leakage current increases with increasing cell size which supports that oxygen migration is the dominant mechanism for the resistive switching behavior observed in RGO based memory cell. The I-V measurements of Al/GO/Al device is provided in supplementary Figure S8, for different cell diameter to support the above data.

**Figure 7:**
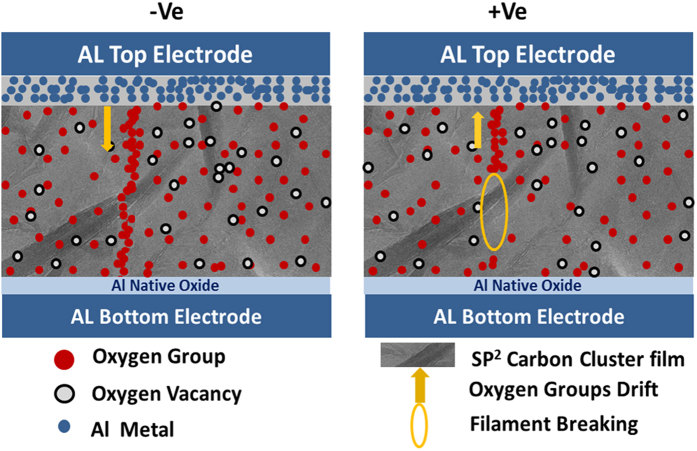
(**a**) Filament formation during SET process when −ve voltage was applied on top electrode. (**b**) Breaking of filament during RESET process when +ve voltage was applied on top electrode.

From the I-V curve, it is seen that the electrode plays a significant role for the better performance of the device. Therefore, the resistive switching behavior of a GO based memory device is studied using different metal electrode such as Al, Cr, and Au. It is interesting to observe that Al/RGO/Al device exhibits a better device performance compared to Cr/RGO/Cr device. However, we could not observe any kind of resistive switching phenomenon in Au/RGO/Au memory device by replacing the electrode with novel metal like Au due to no formation amorphous dielectric layer by the Au electrode (result is not shown here) at the interface. In order to understand the mechanism of resistive switching in RGO based memory devices, different models have been proposed; rupture/formation of conducting filament as well as trapping/detrapping of charge carriers to clarify the bipolar resistive switching. However, by forming conducting filaments mechanism significantly varies for different system. Therefore, creation of the conducting filament in the device during the phenomenon of resistive switching certainly supports by the oxygen vacancy mechanism^18^,^19^ as well as filament formation^20^. The switching mechanism of the Al/GO/Al RRAM can be described by the filament formation and rupture process as follows. Aluminum is one of the most oxidizable metals, hence, oxygen atoms presents in the GO matrix starts diffusing towards the top electrode and starts reacting to the aluminum metal layer because of the higher oxygen affinity of Al as well as the presence oxygen concentration gradient^21^. This route favors the formation of reasonable thick amorphous layer of aluminum oxide at the interface region^22^ by removing oxygen from the adjacent GO layer, and consequently inducing an oxygen vacancy into it. By applying negative polarity to the top Al electrode of the device (Fig. 7a), the oxygen vacancies are filled by oxygen ions diffuse into the dip region of the GO film which favors the growth of local conductive filament to activate “Set” process. As a result, the resistive switching device moves to the lower resistive state. However, during the “Reset” process, (Fig. 7b) these oxygen ions repel back towards the Al/RGO interface and initiate the conducting filament rupture once the device is subjected to the reversal of the polarity (positive). This allows the device to transit into high resistive state. Both resistive switching devices Al/RGO/Al and Cr/RGO/Cr exhibit this phenomenon during the switching process. I-V characteristics of Al/GO/Al memory device at different temperatures are shown in Figure S9.

**Figure 8:**
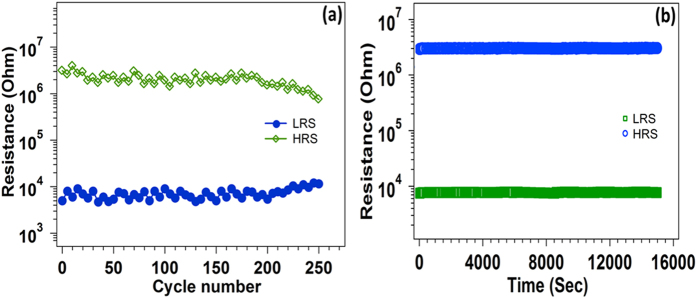
(**a**) Endurance properties and (**b**) retention characteristics of the Al/RGO/Al resistive memory device.

For practical application of the resistive switching device, we investigated the retention and endurance properties of this MIM structure for different cycles and time period and represented in Fig. 8a,b. The performance of the device is extremely good and it maintain a stable HRS and LRS state over many stress cycles having “on/off” ratio ~10^2^. Similarly, it exhibits well retention characteristic without observation of any degradation between two resistance ratio states. We have only represents here the part of the switching cycle and retention, but the device can exhibit few hundred switching cycles as well as many retention periods. The endurance and retention properties indicate that the repeatability and stability of the device is suitable for nonvolatile memory application.

**Figure 9:**
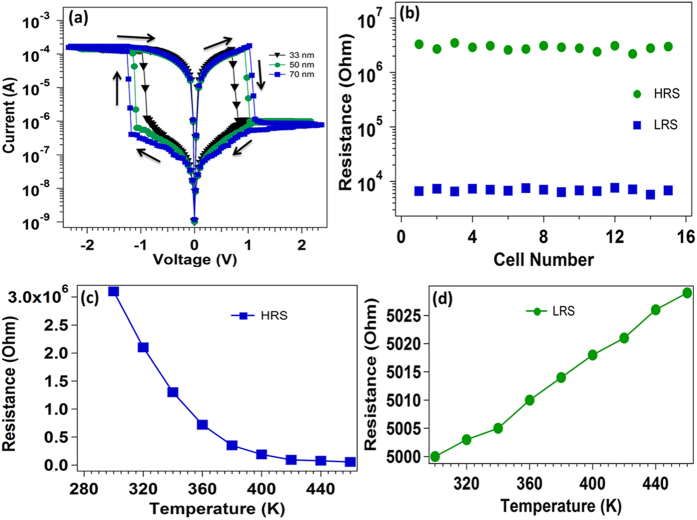
(**a**) GO Thickness dependent resistive switching behavior of Al/GO/Al (**b**) HRS and LRS of Al/GO (33 nm)/Al for different cells (15 number) at different place (**c**) Temperature dependence of resistance of Al/GO (33 nm)/Al device in the HRS (**b**) Temperature dependence of resistance of Al/GO (33 nm)/Al memory device in LRS.

As graphene oxide is one of the promising material for ReRAM application, this material is showing both unipolar as well as bipolar switching depending upon the configuration of the devices. Kim *et al.* observed the unipolar resistive switching behavior of graphene oxide in ITO/GO/ITO structure by applying a forming voltage of (±15 V), which is always needed to make first transition from pristine to LRS. By the application of 0 to +15 V or (−15 V), the device shows the sudden decrease in the current near +13 V (or −11 V). The ITO/RGO/ITO device was thereby switched to its HRS mode. The device then achieved a LRS at approximately ±2 V (VSET) and ±5 V (V_RESET_) when the voltage was swept from zero to a positive (or negative) value^23^. However, our Al/GO/Al device (33 nm of GO) operate at low voltage and shows bipolar switching with V_SET_ and V_RESET_ of less than (±1 V). Furthermore, we study the different GO thickness dependent resistive switching behavior of the device as shown in Fig. 9a. All the device is showing bipolar switching with slowly increase in both V_SET_ and V_RESET_ value with increase in GO layer thickness. However, the LRS and HRS of the of the devices changes marginally. Therefore, it is unlikely that the bulk region of GO contributes to the RS effectively^24^. Most likely, the RS occurs within a spatially confined region. The HRS and LRS from I-V measurement of multiple cells at difference positions of 33 nm GO device is shown in Fig. 9b. The devices performed well in both states, maintaining almost the same ratio over many repetitive cycles. We tested 15 different cells at different place to observe the behavior of resistance in different cell with statistical distribution of LRS and HRS.

From the I-V results, we infer that observation of resistive switching in Al/GO/Al device for different thickness of GO is attributed to the formation/rupture of filament due to oxygen ion movement from top to bottom electrodes under a bias voltage. In case of thin GO layer (33 nm), small negative voltage on the top electrode (<1 V) generates a sufficient amount of electric field that drives the oxygen ion into the GO matrix and form conducting filaments inside the GO layer, and the device reaches the ON state at lower set voltage. However, increase in GO layer thickness required higher voltage to drive the oxygen ion from top to bottom electrode, therefore the device required higher set voltage to reach the LRS^25^,^26^.

Figure 9c,d show temperature dependent resistance state of the device to identify the physical nature of the resistive mechanism in the memory device. The resistance of the device in HRS start decreasing with increase in temperature from 300 to 460 K which is characteristic feature of semiconducting behavior of the device at HRS as depicted in Fig. 9(c). The resistance of the device decreased with increasing temperature in HRS^27^,^28^.

In contrary to semiconducting behavior in HRS, in LRS, the resistance of the device was found to increase linearly with increasing temperature as can be seen in Fig. 9(d), indicating metallic behavior. The metallic conducting behavior in LRS indicates the formation of conducting filaments in GO films^29^.

**Figure 10:**
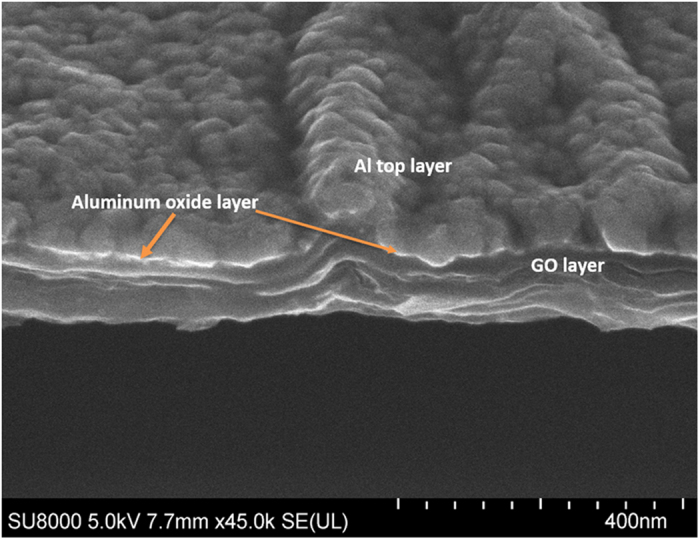
Cross sectional FESEM image of thick GO in Al/GO/Al device. The charging layer shows the formation of Al_2_O_3_ in top Al electrode and GO interface.

Figure 10 shows the cross sectional FESEM image of top Al electrode on GO layer. As Al is recognized as one of the most oxidizable metals redox reaction occurs at the interfaces between the GO layer and Al electrode to form an aluminum oxide (AlOx) layer. In order to identify this amorphous aluminum oxide layer, we performed the cross sectional FESEM by tilting the sample to see the interface. We observed a uniform distribution of top Al metal layer over the GO layers. As amorphous aluminum oxide is highly insulating in nature we observed a charging effect in between the top aluminum layer on layered-like GO film (marked by the arrow). This indicates that aluminum oxide is the initiator for the formation of conducting filaments in GO films as previously proposed^28^.

In summary, the metal/reduced graphene oxide/metal memory device exhibited excellent switching characteristics with on/off ratio of two orders of magnitude and operated at less than 1 V. It is seen that the dominant mechanism for the occurrence of resistive switching behavior is governed by the formation of conducting filament due to Al diffusion and oxygen migration plays a minor role for switching behavior. The Al/RGO/Al device shows a better performance such as lower switching voltages, stable resistance ratio between LRS and HRS as well as good endurance and retention properties which makes this device more favorable for memory application. The studies related to different cell diameter, smaller thickness, and different scan voltages and for different period of time are important findings in this field. The structural, electrical as well as spectroscopic studies ascertain that the device made from the thermally reduced graphene oxide shows more reliability for its use in next generation electronic devices.

## Methods

Graphene oxide was synthesized using modified Hummer’s method from the graphite flakes^30^. Natural graphite powders (2 gm) were oxidized using (KMnO_4_, 7 g) and H_2_SO_4_ (50 ml) at very low temperature 4 °C. KMnO_4_ powder was added slowly to the solution of natural graphite and H_2_SO_4_ and maintained the temperature below 5 °C. The mixture was then continuously stirred at room temperature for about 2 hours after adding more distilled water. Small amount of Hydrogen peroxide (H_2_O_2_, 30 wt%) was added slowly to the solution in order to facilitate the dissolving of any unreacted KMnO_4_ . Then the solution was centrifuged and repeatedly washed with warm water (70 °C) to obtain the GO solution. The solutions are sonicated for 30 minutes and collected the suspended GO solution followed by drying to get GO powder. GO powders were heated at 50, 100, 150 °C for 5 hours in a vacuum oven for thermal reduction.

The resistive switching memory cell consists of metal/insulator/metal (MIM) structure, using thermally (100 °C) reduced graphene oxide for 5 hours as an insulator layer. However, further thermal reduction of GO at higher temperature causes more difficulty to make a well dispersed solution for spin casting. The solution is comprised of H_2_O, reduced graphene oxide, and methanol in 2 mg/ml concentration. Final solution is sonicated for 4 hours to obtain uniform dispersion of RGO in H_2_O and methanol and centrifuged to 5000 rpm to remove any large flakes of reduced graphene oxide from the solution. Cr and Au metals were deposited in high vacuum (10^−8^ torr) using electron beam evaporator and Al was deposited using thermal evaporator on commercially available glass substrates to make bottom electrode of the device. The metal coated glass substrates were exposed to the ultraviolet light source in order to improve the adhesion of GO into the bottom electrode. This GO solution was spin coated onto the bottom electrode with blowing N_2_ to get the desired thickness of the films resulting in formation of few of layers of graphene coating that comprises of a stack of individual GO sheets onto the substrate. By blowing N_2_ onto the electrode surface improves the uniformity of GO layer as well as removes the solvent. Samples were allowed to dry completely at room temperature, followed by heating it at 70 °C for 1 hour in vacuum oven. Top electrodes of similar metal were deposited through a shadow mask of different diameter over the GO film. Thus, the device structure formed was metal/GO/metal on glass substrate with thickness of each top and bottom electrodes of 50 nm.

Thickness of the sample was measured using a profilometer (Make-Bruker, DektakXT). XRD spectra were collected using Rigaku Diffractometer. Photoluminescence spectra were collected using Hitachi F-7000 spectrophotometer. Raman spectrum was collected in a micro-Raman spectrophotometer system (Make-Horiba Scientific, Labram HR evolution) with an exciting laser wavelength of 785 nm having a small diameter of laser beam, approximately 0.5 mm. The electrical measurements were performed using a Keithley 4200-SCS semiconductor characterization system with voltage sweeping mode. During the measurement, a bias voltage was applied between the top and bottom electrodes with the latter being grounded. The surface morphology of the GO was acquired using Hitachi SU-8010 field emission SEM, and TEM images and selected area electron diffraction (SAED) patterns were obtained at 200 kV on a JEOL 2000FX with a Gatan SC-1000 Orius CCD camera.

## Additional Information

**How to cite this article**: Pradhan, S. K. *et al.* Resistive switching behavior of reduced graphene oxide memory cells for low power nonvolatile device application. *Sci. Rep.* **6**, 26763; doi: 10.1038/srep26763 (2016).

## Supplementary Material

Supplementary Information
