# Supplementary material for: Resistive switching behavior of reduced graphene oxide memory cells for low power nonvolatile device application
Source: Sci Rep. 2016 May 31;6:26763. doi: 10.1038/srep26763 (PMC4886213; doi:10.1038/srep26763)
Supplement: Supplementary Information [file srep26763-s1.pdf]

Resistive switching behavior of reduced graphene oxide memory cells for low power nonvolatile device application

S. K. Pradhan, Bo, Xiao, S. Mishra, A. Killam, A. K. Pradhan

Center for Materials Research, Norfolk State University, 700 Park Ave., Norfolk, VA 23504

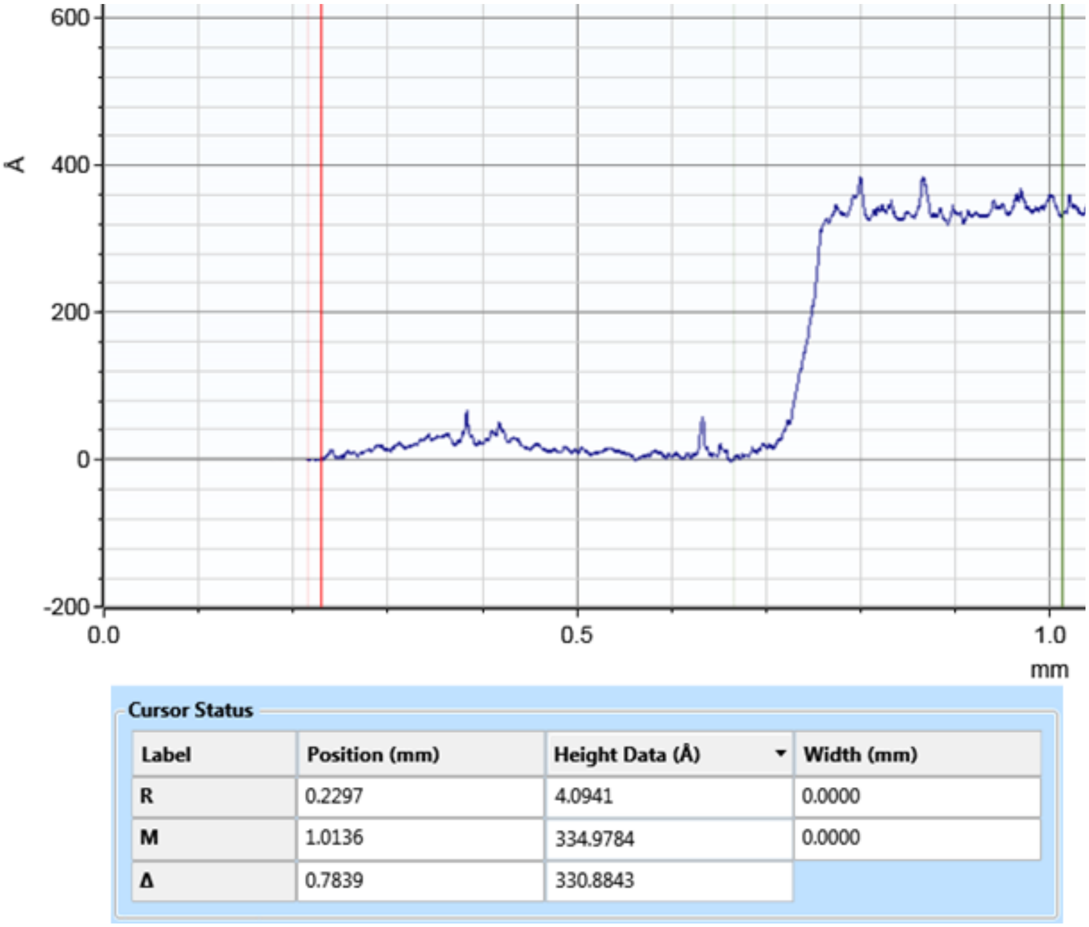

Label	Position (mm)	Height Data (Å)	Width (mm)
R	0.2297	4.0941	0.0000
M	1.0136	334.9784	0.0000
Δ	0.7839	330.8843	

Figure S1. Thickness of the RGO film measured using profilometer.

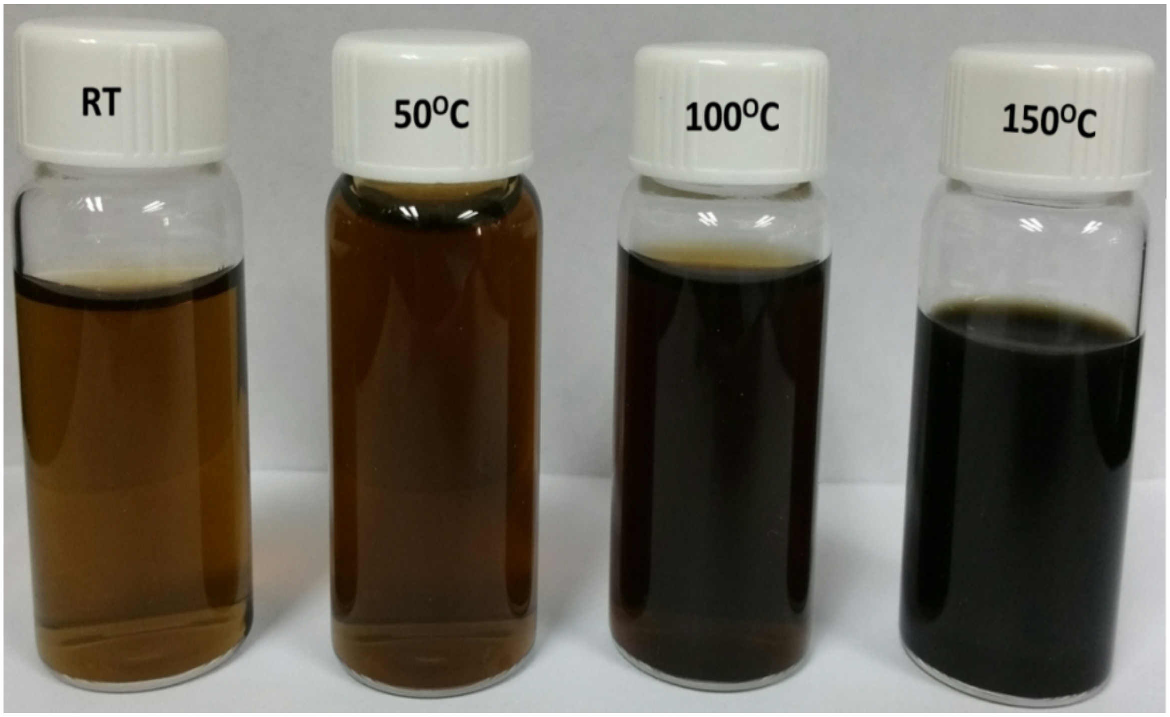

Figure S2. Visual image of thermal reduced GO dispersion in water molecule at different annealing temperature for 5 hours.

Figure S2 represents the visual image of GO solution (left to right) for different thermal annealing temperature. The clear brown color of GO suspension represents the highly oxidized nature of GO at room temperature. On increase in temperature from 25°C to 150°C, the color of the GO solution turns towards black which is a characteristic feature of thermally reduced graphene oxide.

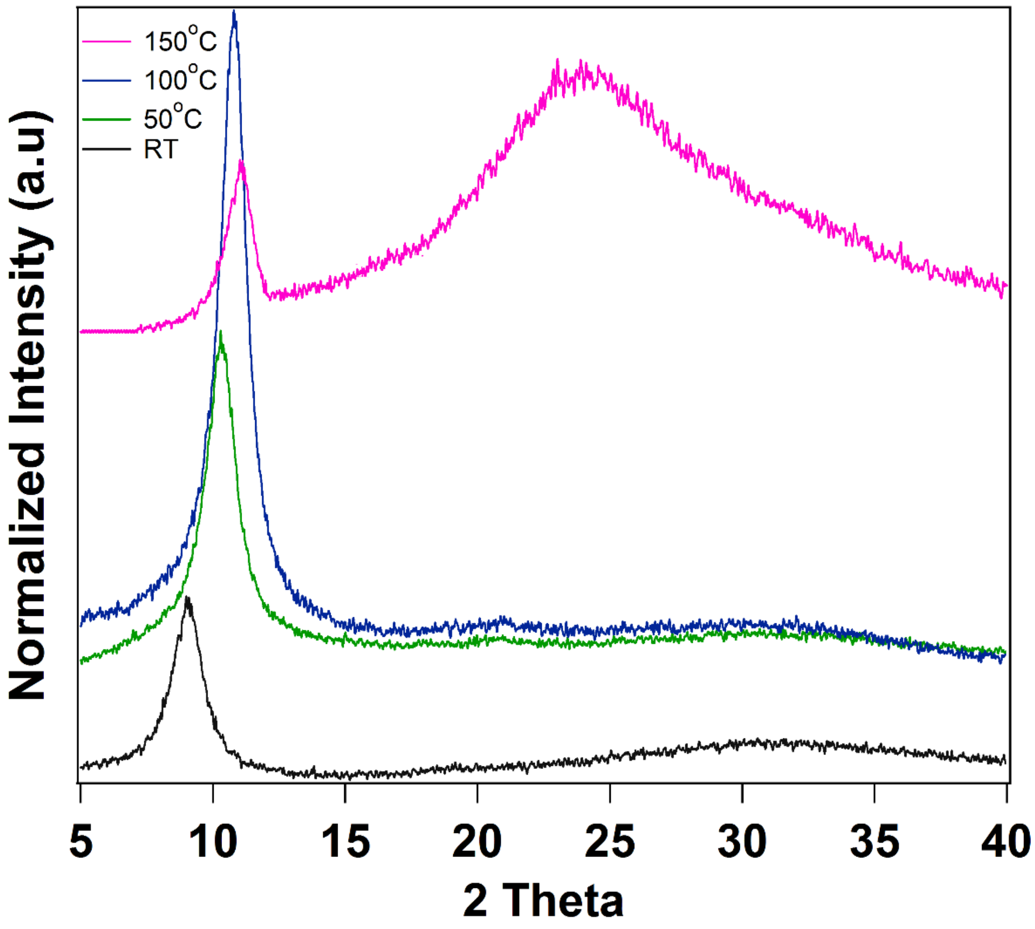

Figure S3. XRD structure of Graphene Oxide reduced at different temperature.

Temperature dependent XRD pattern of GO from room temperature to 150°C are shown in figure S3. At room temperature the GO shows a peak (002) at 2θ value close to 9°. With gradual increase in temperature the (002) peak of GO starts shifting towards higher 2θ value with a variance of intensity as well as full width half maxima (FWHM). This is due to drastic vaporization of intercalated water molecules present in the GO [1]. On further increase in thermal reduction temperature of GO to 150°C, there is a formation of new broad peaks around ~ 2θ = 24°C in the XRD pattern of the sample.

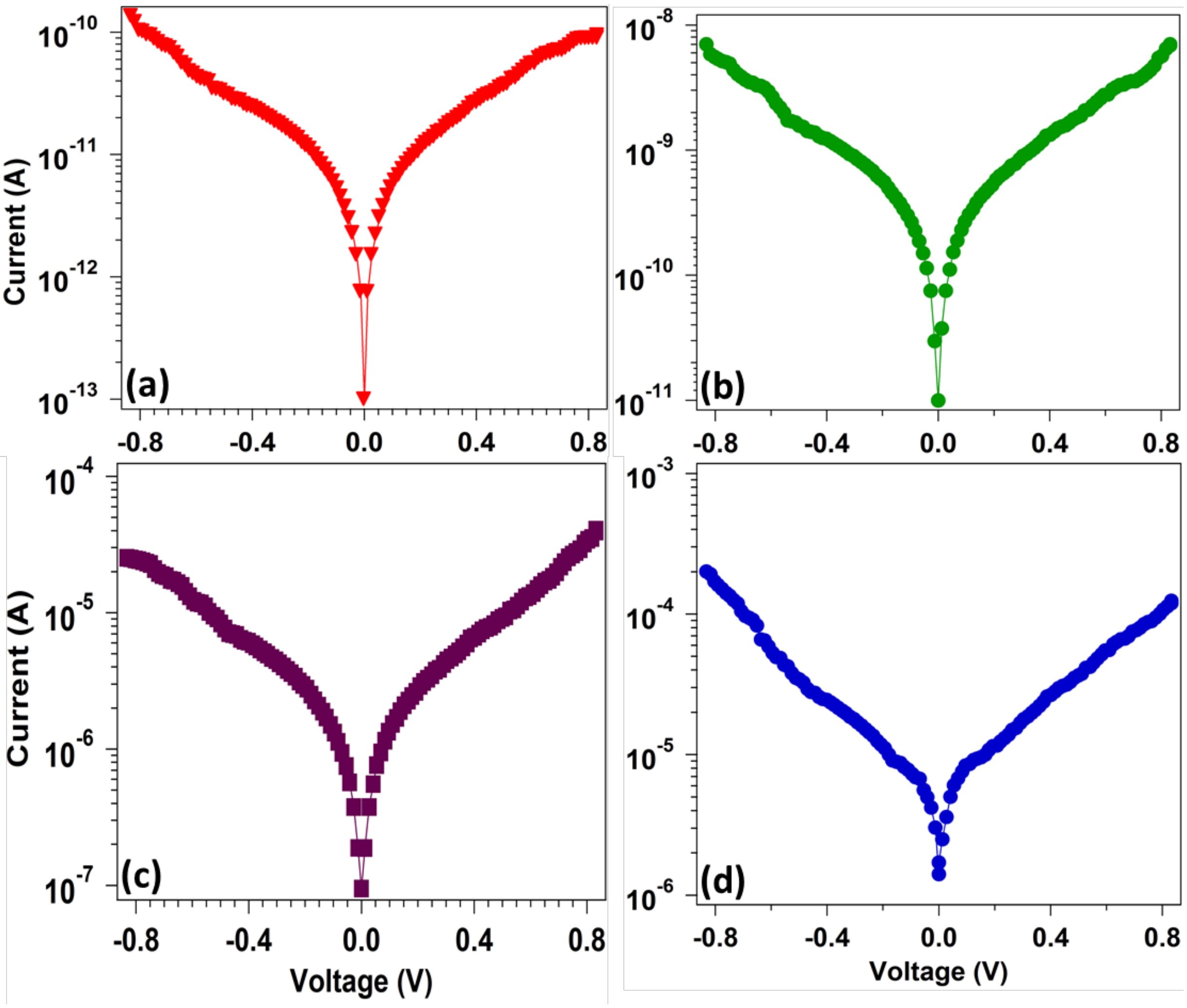

Figure S4. I-V characteristic of Graphene Oxide reduced at different temperature (a) Room Temperature (b) 50°C (c) 100°C and (d) 150°C.

Figure S4 represents the I-V characteristic of the GO and thermally RGO at different reduction temperature. From the graph it is observed that as prepared graphene oxide is highly insulating in nature and the value of current is in the range of nano ampere to pico ampere range due to the presence of oxygen and other function group during the chemically oxidation process. However, the conductivity of the sample starts increasing by the heat treatment because of the removal of residual water molecule as well as other

chemical groups. However, further increasing in thermal reduction temperature to 150°C enhances the conductivity of RGO and the value of current observed in the sample is in the range of few milliampere to micro ampere. This is further confirmed by FTIR measurement.

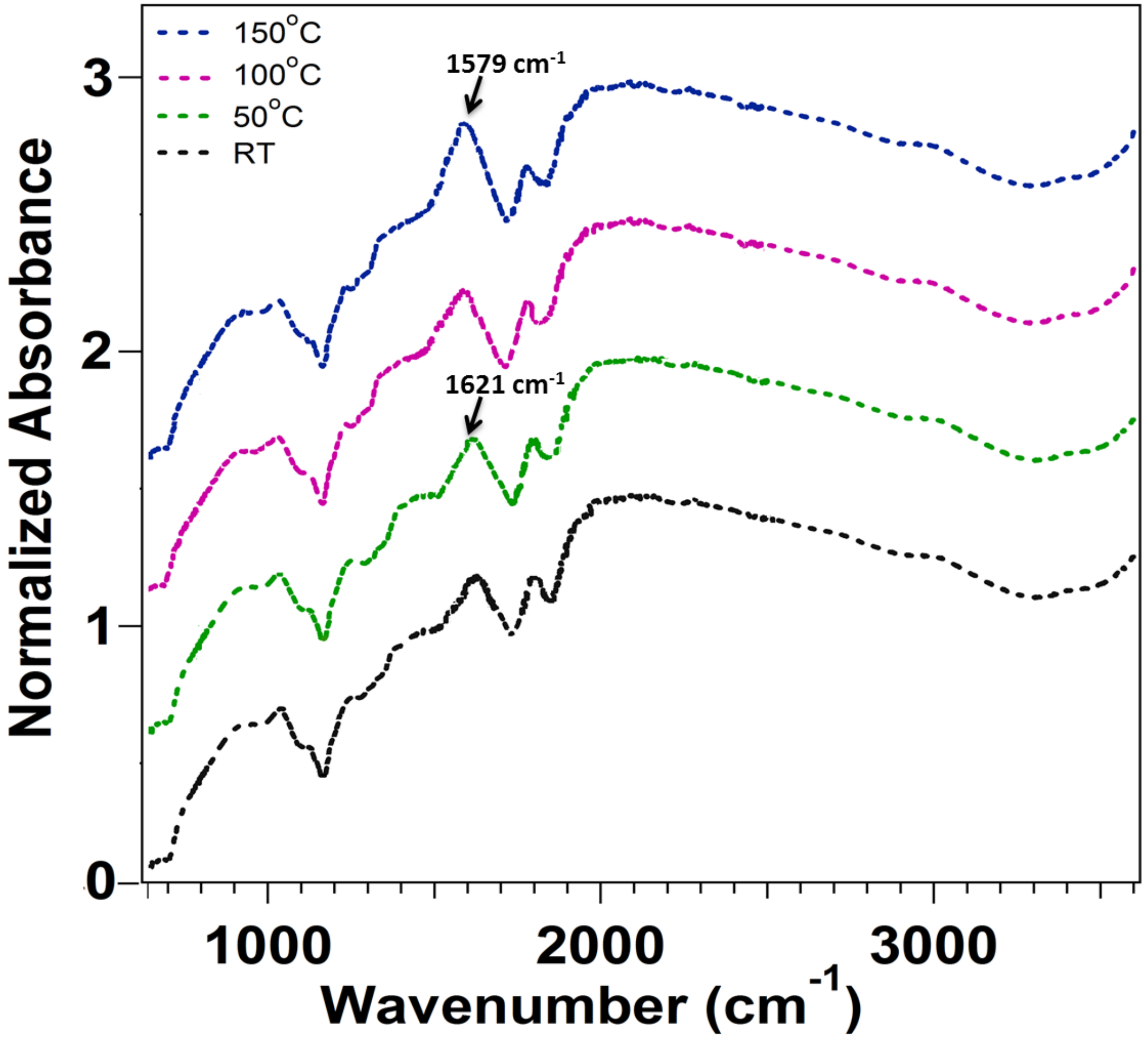

Figure S5. FTIR spectra of Graphene Oxide reduced at different temperature.

Fourier transform infrared spectroscopy (FTIR) is suitable technique to study the visible absorption of annealed GO sample as shown in figure S5. The obtained FTIR spectrum of RT clearly indicates the existence of oxygen functional group and again confirms no major removal of oxygen functional group from the basal plane of graphene and the absorption peak of 1621 cm^{-1} signify the C=C skeletal vibration of graphitic domain or the deformation vibration of intercalated water molecules remains more or less unchanged. even at the low annealing temperature of ($50^{o}C$) GO. However, GO reduced GO annealed at $100^{o}C$, the peak intensity decrease and is due to loss of intercalated water molecule with the observation of peak at 1579 cm^{-1}, which is attributed to the formation of prominent graphitic domain in GO, consistent with the other reports [2-4].

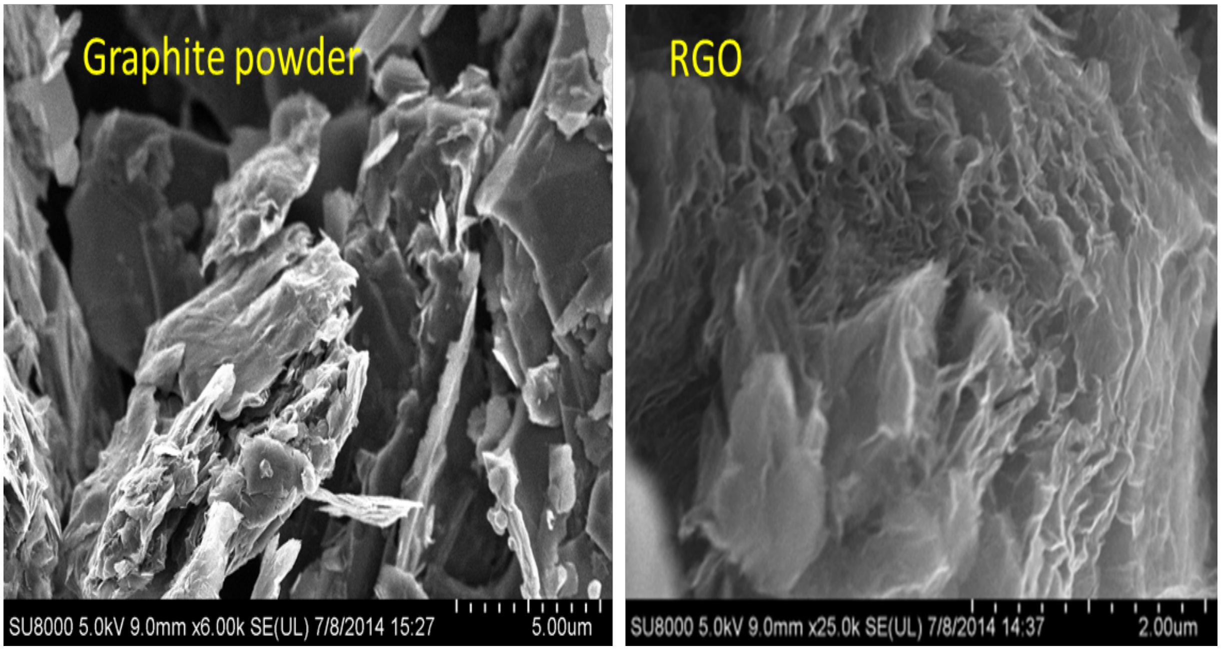

Figure S6. FESEM images of (a) Graphite powder (b) reduced graphene oxide at $150^{o}C$.

FESEM image of graphite powder and RGO powders are shown in figure S6. The graphite powder does not show any kind of graphene paper or sheet due to its compact structure. However, RGO at 150°C shows beautiful images of GO sheets/papers like petals of a flower. In other way, we could not obtained any contrast FESEM image of as prepared GO and low temperature thermally RGO due to the changing effect and do not put the images here for comparison.

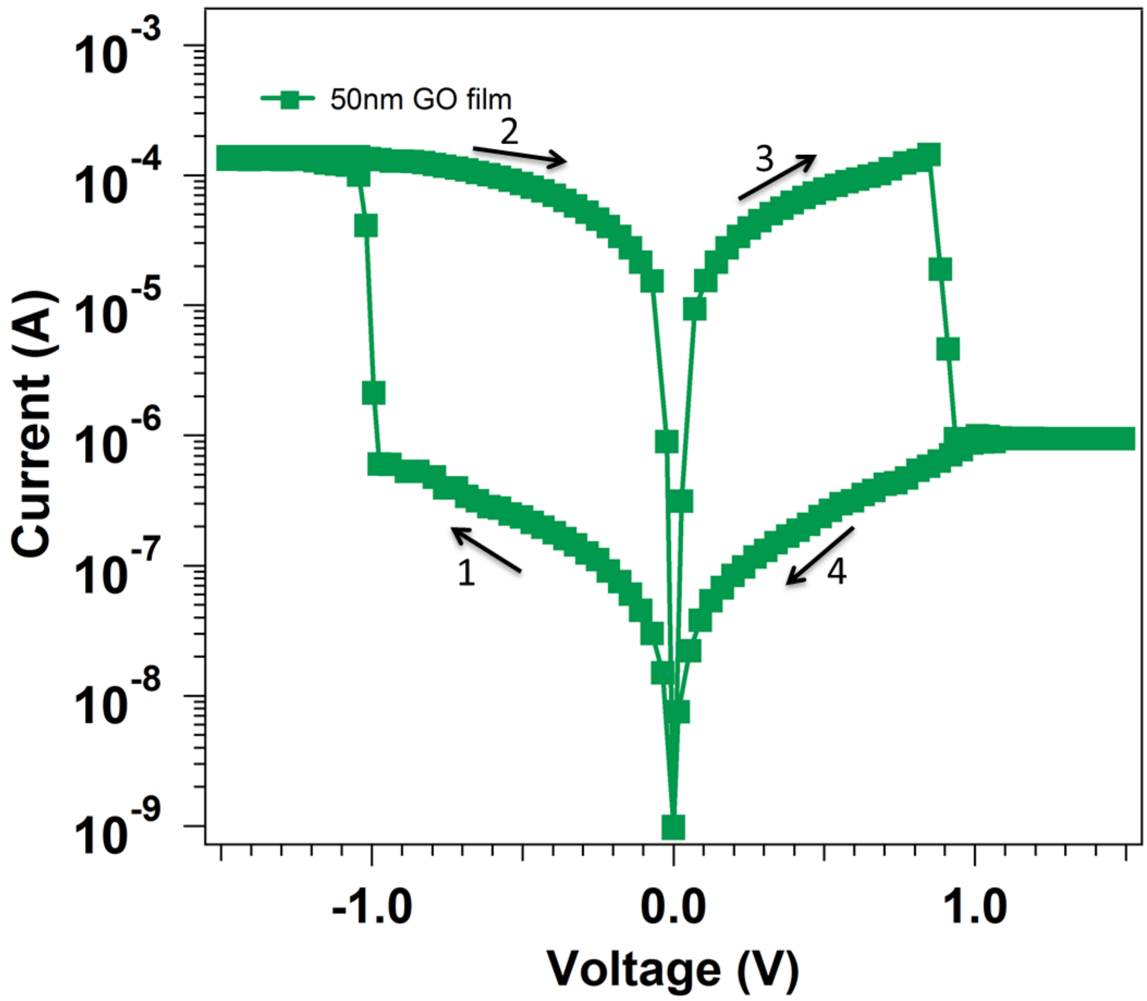

Figure S7. shows the *I-V* characteristics of Al/GO/Al memory cells using GO film thicknesses of 50 nm

Figure S7 shows the *I-V* characteristics of Al/GO/Al memory cells using GO film thicknesses of 50 nm. It is observed that, the turn on voltage strongly depends on the GO film thickness and increases with increase of GO layer, indicating that the electric field inside the bulk GO is the controlling factor of the switching behavior. However, no variation of HRS resistance and LRS resistance of the device is observed with increase in GO film thickness to 50 nm. Hence, the thickness-insensitive property of HRS resistance and LRS resistance indicates that once the turn on voltage state is completed, it is unlikely that the bulk region of GO contributes to the resistive switching effectively. This also underpins that the switching phenomenon occurs at the interface rather than the bulk region.

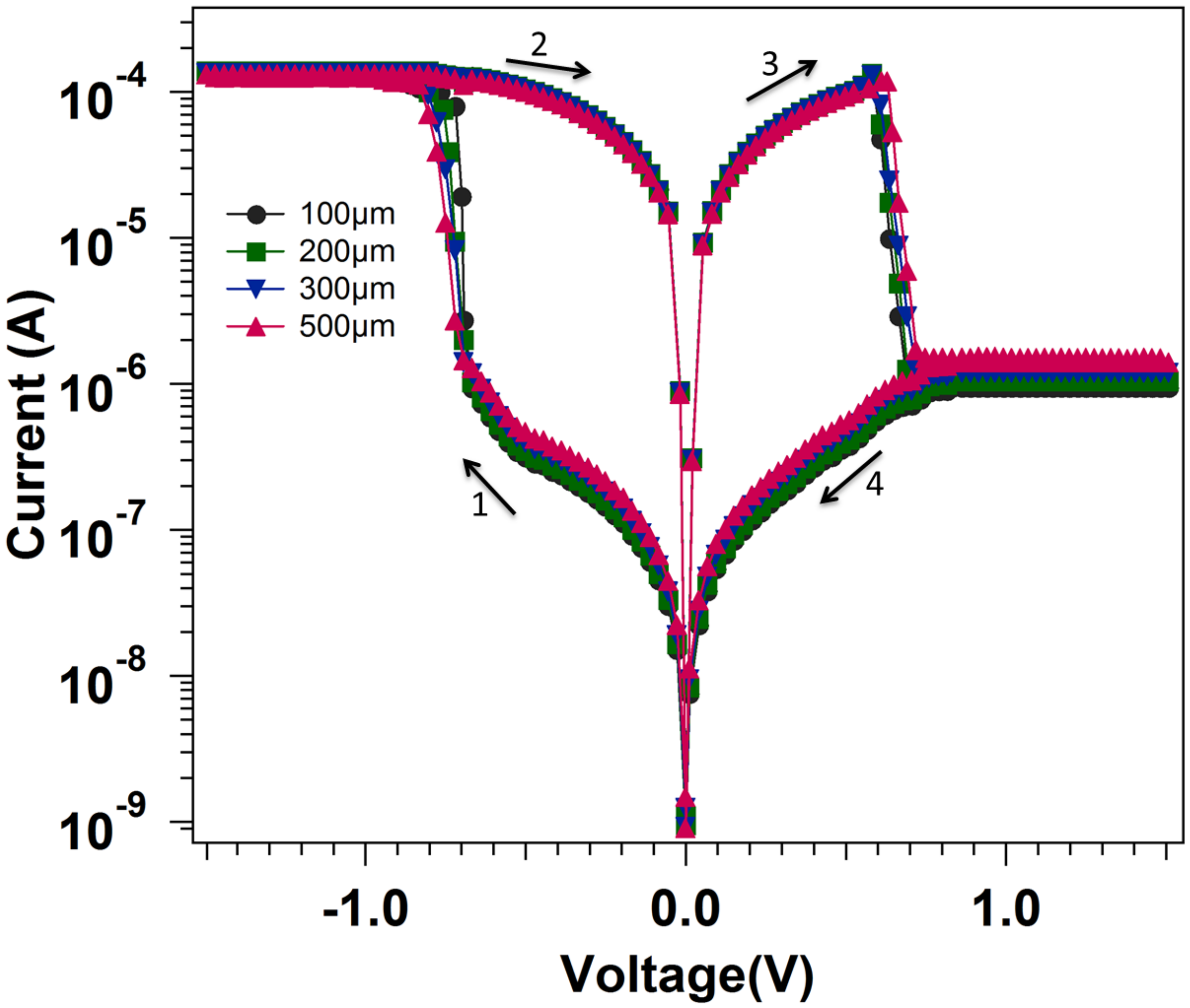

Figure S8 shows the I-V measurement of Al/GO/Al device for different cell diameter.

Figure S8 shows the I-V measurement of Al/GO/Al device for different cell diameter. The current in HRS is change more as compare to LRS when the electrode area is increased, resulting a lower HRS/LRS resistance ratio. This kind of resistive switching behavior prevails the filamentary type conduction occurs in the device.

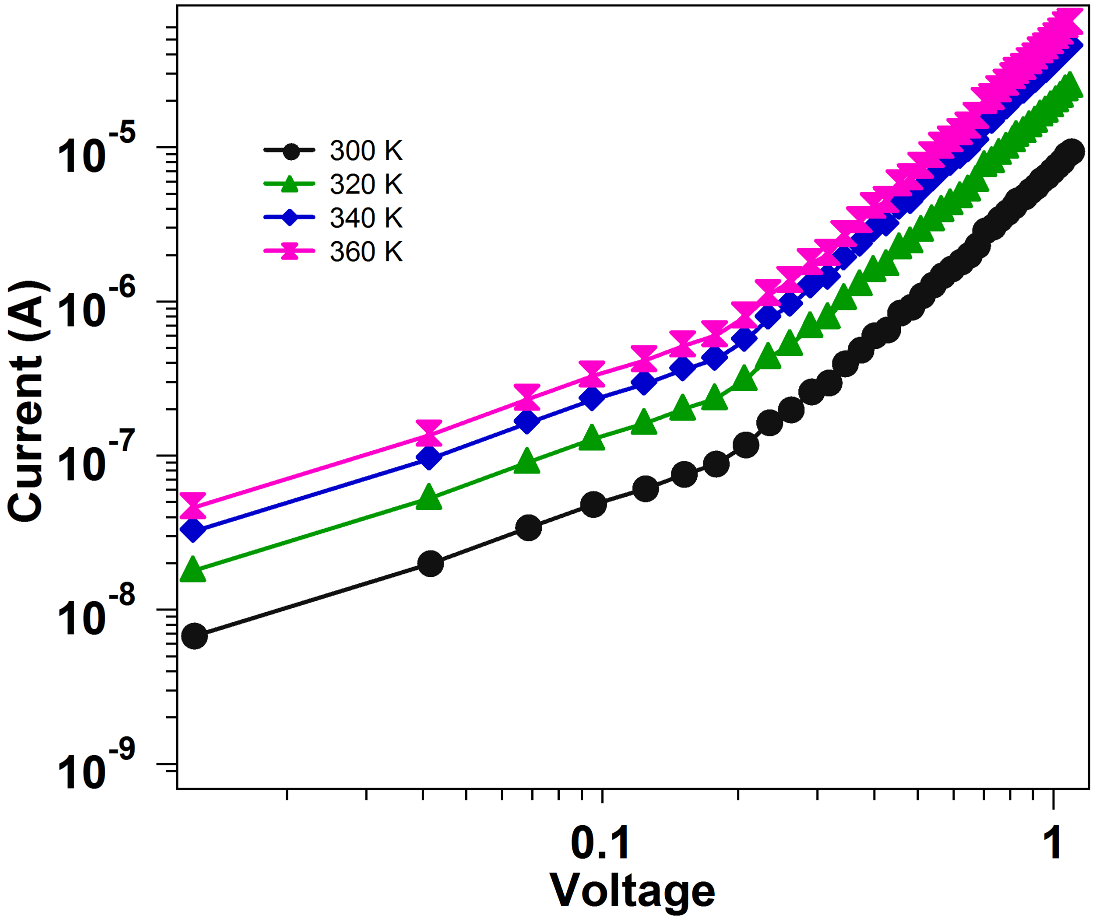

Figure S9. Ln (I)- ln (V) characteristics of Al/GO/Al memory device at different temperatures.

Figure S9 shows the ln (I)- ln (V) characteristics of Al/GO/Al memory device at different temperatures at the sweeping voltage step of 0.03V/s. It can be seen that the turn on voltage decreases with increasing temperature, suggesting that the thermal generation of the carrier increases with temperature can provide enough energy for the oxygen ions to jump out from its original lattice site resulting in formation of an oxygen vacancy, and the

motion ability of oxygen vacancy directly proportional to the temperature. Because the energy needed for formation and migration of the oxygen vacancy are both provided from the external environment. The observed results in the low voltage region indicate that there might be some preexisted tiny defects such as oxygen vacancies inside the pristine GO thin films, thus contributes the leakage. So, the conducting behavior follows the hopping conduction, which jumps between the discrete defects inside the GO films.

References

1. Jeong, H. K.; Lee, Y. P.; Jin, M. H.; Kim, E. S.; Bae, J. J.; Lee, Y. H. Thermal stability of graphite oxide. *Chem. Phys.Lett.* **2009**, *470*, 255−258.

2. Bagri, A. Mattevi, C.; Acik, M.; Chabal, Y. J.; Chhowalla M.; Shenoy Vivek B. Structural evolution during the reduction of chemically derived graphene oxide. *Nature Chem.* **2010**, 2, 581–587.

3. Fan, X. Peng, W.; Li, Y.; Li, X.; Wang, S.; Zhang, G.; Zhang F. Deoxygenation of exfoliated graphite oxide under alkaline conditions: a green route to graphene preparation. *Adv. Mater*. **2008**, *20*, 4490–4493.

4. Rourke, J. P.; Pandey, P. A.; Moore, J. J.; Bates, M.; Kinloch, I. A.; Young, R. J.; Neil, R. W. The real graphene oxide revealed: stripping the oxidative debris from the graphene-like sheets. *Angew. Chem. Int. Ed.* **2011**, *50*, 3173–3177.
